# Quality of chronic disease care in general practice: the development and validation of a provider interview tool

**DOI:** 10.1186/1471-2296-8-21

**Published:** 2007-04-19

**Authors:** Judith Proudfoot, Upali W Jayasinghe, Fernando Infante, Justin Beilby, Cheryl Amoroso, Gawaine Powell Davies, Jane Grimm, Christine Holton, Tanya Bubner, Mark Harris

**Affiliations:** 1 Centre for Primary Health Care and Equity, School of Public Health & Community Medicine, University of New South Wales, Sydney, NSW 2052, Australia; 2 Department of General Practice, The University of Adelaide, SA 5005, Australia

## Abstract

**Background:**

This article describes the development and psychometric evaluation of an interview instrument to assess provider-reported quality of general practice care for patients with diabetes, cardiovascular disease and asthma – the Australian General Practice Clinical Care Interview (GPCCI).

**Methods:**

We administered the GPCCI to 28 general practitioners (family physicians) in 10 general practices. We conducted an item analysis and assessed the internal consistency of the instrument. We next assessed the quality of care recorded in the medical records of 462 of the general practitioners' patients with Type 2 diabetes, ischaemic heart disease/hypertension and/or moderate to severe asthma. This was then compared with results of the GPCCI for each general practice.

**Results:**

Good internal consistency was found for the overall GPCCI (Cronbach's alpha = 0.75). As far as the separate sub-scales were concerned, diabetes had good internal consistency (0.76) but the internal consistency of the heart disease and asthma subscales was not strong (0.49 and 0.16 respectively). There was high inter-rater reliability of the adjusted scores of data extracted from patients' medical notes for each of the three conditions. Correlations of the overall GPCCI and patients' medical notes audit, combined across the three conditions and aggregated to practice level, showed that a strong relationship (r = 0.84, p = 0.003) existed between the two indices of clinical care.

**Conclusion:**

This study suggests that the GPCCI has good internal consistency and concurrent validity with patients' medical records in Australian general practice and warrants further evaluation of its properties, validity and utility.

## Background

Chronic conditions such as diabetes, cardiovascular disease, asthma and depression impose a significant challenge to health systems throughout the world contributing 47% to the global burden of disease[1]. In Australia, such conditions account for 70% of the total burden of disease[2]. The majority of chronic disease care takes place within general practice and one in four general practice consultations are for problems associated with such illnesses[3]. However, in Australia [4-6] and in other countries [7-9], the care provided in this setting is often sub-optimal. Several factors contribute. The historical focus on organisational systems to facilitate acute care in general practice, which, with its predominant episodic nature, short consultations and little emphasis on the patient's role as partner in the care process, hampers the capacity of general practitioners to undertake high quality, sustainable chronic disease care[10]. Many chronically ill patients have multiple conditions as well as complex personal and social circumstances, yet clinical and social information is often poorly recorded, even when general practitioners have a special interest in the management of a specific chronic illness[11]. In particular, items are often missing, and frequently it is not known whether the absence of details about an activity, such as an examination or a test, is because it has not been performed, or, on the other hand, it has been performed but not recorded. Further, information about care relating to the subjective aspects of a patient's condition, those aspects which constitute the patient's experience of their 'chronic illness'[12] is often poorly captured.

There is an urgent need for systems within general practices to deliver information about the care of patients with multiple conditions in a more comprehensive, systematic and efficient way, in order to facilitate quality assessment and, ultimately, quality improvement. However, attempts to date to conduct simultaneous quality assessment across multiple conditions have been shown to be problematic[13]. In addition to the poor quality of data recorded, low prevalence of some items of care and unreliable data extraction processes are common and they prevent reliable assessment. The solution, according to some professionals, is to develop more efficient and reliable data extraction methods[14]. However, such an approach does not address the variable quality of data in patients' medical notes and the large investment of resources needed to extract those data reliably. Nor does it capture the subjective aspects of quality of care, where the general practitioner knows the patient's personal circumstances and adjusts care accordingly.

In this paper, we report on a new instrument, a structured interview schedule that provides simultaneous assessment of provider-reported quality across three chronic conditions within a general practice setting. The main objectives of the paper are twofold: (a) to describe the development and characteristics of the Australian General Practice Clinical Care Interview; and (b) to establish the properties of the instrument, including its reliability and validity.

## Methods

### Development and description of the General Practice Clinical Care Interview

Using Australian published evidence-based clinical guidelines containing quality indicators for Type II diabetes, asthma and ischaemic heart disease/hypertension [15-18], we developed the Australian General Practice Clinical Care Interview (GPCCI), a structured interview instrument to assess the provider-reported care delivered by general practitioners to patients with Type II diabetes, moderate to severe asthma and/or ischaemic heart disease/hypertension. A detailed mapping of the guidelines against the GPCCI is in Appendix I [see Additional file 1]. Experts who were involved in drafting or reviewing the guidelines reviewed and commented on the GPCCI items. The GPCCI was then piloted with 5 general practitioners who were asked to comment on the clarity and comprehension of items.

To minimise the opportunity for general practitioners to answer hypothetically (that is, based on their knowledge of what constitutes best practice) and to capture instead what they actually do on a day-to-day basis, we anchored some of our questions to the general practitioners' specific patients (e.g. 3 newly diagnosed patients with hypertension, 3 patients with Type II diabetes who recently had their HbA1C outside the target range of 7%) and we followed up with questions about whether this care was representative of the care they usually provided to such patients, and if not, how it differed. It also enabled us to capture some of the subjective aspects of quality of care, where the general practitioner knows the patient's personal circumstances and adjusts care accordingly. Other questions asked general practitioners to indicate whether they had performed certain quality functions and to estimate the proportion of their patients who had received different aspects of care.

Four key components of care were assessed for each of the three conditions, as defined by the clinical guidelines:

• case finding, which includes the identification of patients at risk of the condition and methods of screening and diagnosis. Item example: *How do you identify patients for assessment of cardiovascular risk factors?*

• assessment of the key behavioural and physiological variables and the early detection of complications of the condition. Item example: *What proportion of your adult patients with moderate to severe asthma were offered a review of their smoking status in the last 6 months?*

• patient education for self management. Item example: *Thinking of your newly diagnosed patients with Type 2 diabetes, please jot down the initials or a description of 3 of them. Was self-management education provided to these patients? If yes, who provided it and what did it entail? Was the education provided to these 3 patients typical of what you normally do with newly diagnosed diabetes patients? If not, why not and what is typical?*

• ongoing care, which includes how patients who are poorly controlled are managed (including further assessment, changes to treatment and referral), the use of evidenced based guidelines, support for self management, patient-held records, care planning, follow up and monitoring. Item example: *How do you follow up people with asthma who do not attend their appointments?*

These four components represent key clinical activities of general practice in caring for patients with chronic disease in the Australian health system[19]. The interview schedule consists of 56 questions. Higher clinical scores (max = 78) reflect better clinical care.

### Evaluation of the General Practice Clinical Care Interview

We evaluated the psychometric properties of the GPCCI in two ways. First, we conducted an item analysis and tested the internal consistency of the overall scale using Cronbach's alpha[20]. Then we validated our GPCCI against the care recorded in the medical records of the general practitioners' patients with Type 2 diabetes, ischaemic heart disease/hypertension and moderate to severe asthma.

### Sample

The study was conducted within five Divisions of General Practice in two Australian states, New South Wales and South Australia. The Divisions of General Practice issued invitations to participate in the study to their constituent general practices. Ten general practices agreed to take part, representing a mix of practice types, including solo practitioner (3), group (6) and corporate (1). Six of the practices were in New South Wales, the remaining four were in South Australia.

General practitioners (family physicians) within each of the practices were invited to be interviewed. A minimum sample of 50% of the general practitioners per practice was set in order to ensure representativeness of the data. Eligible patients at the same general practices were invited to participate in the study. Patients were eligible if they had one or more of the target conditions (Type 2 diabetes, moderate to severe asthma and ischaemic heart disease/hypertension, as diagnosed by their general practitioner), aged 18–85 years old, able to read English sufficiently to understand the information and consent forms. Patients were recruited in strict chronological order as they presented at the participating practices for a consultation. The number of patients with each condition reviewed within each practice is in Appendix II [see Additional file 2]. This shows that there was some variability between practices in the number of patients with each condition recruited, however these differences were not statistically significant.

### Ethics

The study was approved by the Human Research Ethics Committees of the University of New South Wales and the University of Adelaide. Participating general practitioners and patients received information on the study and completed a written consent form prior to participation. Practices were compensated for the time of staff participating in the study and patients were entered in a draw for three prizes.

### Data collection and analysis

#### General Practice Clinical Care Interview

The GPCCIs were conducted in the offices of the twenty-eight participating general practitioners. Due to the geographic distance between surgeries, two researchers in each state carried out the interviews, one researcher to one general practitioner interviewee. The researchers were unknown to the general practitioners. Psychometric analyses of the resultant data were conducted, including calculation of the internal consistency of the scale.

#### Medical record audit

Three data extraction proformas, one each for asthma, Type II diabetes and ischaemic heart disease/hypertension, were developed from the same evidence-based guidelines [15-18] used to develop the GPCCI. The proformas were used to extract information from patients' medical notes and were scored for analysis. Maximum points possible were 14 (diabetes), 11 (asthma) and 9 (heart disease), creating an overall total of 34. Higher scores indicated better clinical care. Five raters extracted the data from the medical records in general practices across the two states, according to a strict protocol. The five raters were post-graduate researchers with experience in conducting research in general practice and clinical experience in chronic disease management.

We verified the reliability of the ratings by analysing the data extracted from 11 patient records (three Type II diabetes, three IHD/hypertension, five asthma) by all five raters. First, single rater reliabilities (or intra-class correlations) were derived from analysis of variance. Single rater reliability is defined by Marsh & Ball[22] as the correlation between two independent assessments of the same subject. One-way ANOVAs were constructed for individual items for each chronic disease. The results were used to assess the strength of agreement between raters for each item in each scale. Using the Spearman-Brown equation, we then calculated reliabilities for the 5 raters for each item.

We removed items that had the same scores from all raters (reliability cannot be calculated) and then conducted a reliability analysis for the adjusted total scores for each chronic disease.

#### Analysis of the correlation between GPCCI and medical record audit

A comparison of the data items and coding for the GPCCI and each of the medical record audits (for Asthma, Diabetes and IHD/Hypertension) and their distributional characteristics is in Appendix III [see Additional file 3]. This shows that the proportion of codes for each element was similar between the GPCCI and medical record audit. A series of Pearson Product Moment Correlation analyses were performed to ascertain the concurrent validity of the GPCCI in relation to the medical record audit. First we computed the correlations separately for the three disease groups aggregated to practice level, to ascertain whether the interview rating scale had stronger validity as three separate scales. Then, for comparison, we conducted the correlations on the total GPCCI, combined across the three conditions.

All analyses were carried out using SPSS version 12.0.1 for Windows[23].

## Results

Twenty-eight general practitioners (68% male, mean age 48.8 years [SD 10.1], mean number of years practicing as a general practitioner 20.5 [SD10.8]) consented to participate in the study. The GPCCI took 35 minutes to administer, and up to 45 minutes if a general practitioner had difficulty thinking of specific patients and had to consult his/her records. Four hundred and sixty-two of their patients with Type II diabetes, ischaemic heart disease/hypertension and/or moderate to severe asthma (49% male) agreed to have their medical notes reviewed.

The results are presented in the order of the analyses undertaken: (i) internal homogeneity of the GPCCI; (ii) inter-rater reliability analysis of the medical record audit, and (iii) correlation of GPCCI and medical record audit.

The internal consistency of scale determined by Cronbach's alpha was α = 0.75 for the overall scale, compared with α = 0.76 for the diabetes sub-scale, α = 0.49 for the heart disease and α = 0.16 for the asthma subscale. The latter coefficients are below the 0.7 cut-off recommended by Nunnally[24]. Descriptive statistics for the GPCCI are presented in Table 1.

**Table 1:** Descriptive Statistics for the GPCCI and Medical Notes Audit (n = 10 practices)

	GPCCI		Medical Notes Audit		CCI/Notes audit
	Max Possible	Mean score (95% CI)	Max Possible	Mean adjusted score (95% CI)	Pearson Correlation Coefficient (p)

**Asthma**	24	10.4 (8.0–12.7)	11	3.3 (2.9–3.8)	0.74 (0.015)
**Diabetes**	30	18.1 (15.5–20.7)	14	7.8 (6.8–8.8)	0.89 (0.001)
**IHD/Hypertension**	24	11.6 (10.1–13.1)	9	4.6 (4.3–4.8)	0.68 (0.031)
**Total**	78	40.0 (34.0–46.0)	34	15.7 (14.1–17.3)	0.84 (0.003)

Table 1 also presents descriptive statistics of the medical record audit, against which the GPCCI was validated. The inter-rater reliability estimates of the adjusted total scores of the data extracted from patients medical notes are summarised in Table 2 for each condition (Type II diabetes, asthma, IHD/hypertension). The analysis for asthma included five patients notes assessed by five raters, whilst the analysis for diabetes and IHD utilised the notes of three patients each assessed by five raters. The results demonstrate that there was strong inter-rater reliability on the adjusted total scores of data extracted from patients medical notes for all three conditions (r = 0.88, r = 0.92, r = 0.90 for diabetes, asthma and heart disease respectively).

**Table 2:** Reliabilities for adjusted total scores

RATING	r_11_	r_22_	r_33_	r_44_	**r_55_**
Diabetes	0.59	0.74	0.81	0.85	**0.88**
Asthma	0.71	0.83	0.88	0.91	**0.92**
IHD	0.63	0.77	0.84	0.87	**0.90**

r_11 _is the single rater reliability and r_kk _is the reliability of k raters

To ascertain whether the GPCCI possesses concurrent validity, a series of Pearson Product Moment Correlation analyses were computed on the GPCCI and patients' medical notes from the same practices. First we correlated the three disease sub-scales separately (diabetes, asthma, heart disease) with the equivalent section of the medical notes to determine whether the GPCCI was more valid as three separate instruments; then we correlated the overall scale. The results indicated that a correlation between the GPCCI and notes audits existed both at the level of the disease specific sub-scales (diabetes r = 0.74, asthma r = 0.89, heart disease/hypertension r = 0.0.68), and also at the level of the overall scale (r = 0.84) (Table 2).

## Discussion

Continuous monitoring and evaluation of the processes of chronic disease management is necessary for constant revision and quality improvement. However, without reliable, sensitive and valid procedures for measuring care, quality initiatives will not succeed[25]. In the UK, the recently-implemented plan to improve quality across the health system has been accompanied by central infrastructure development (the Modernisation Agency, the Commission for Health Improvement, the National Institute of Clinical Excellence), initiatives such as the National Service Frameworks, National Performance Assessment Framework, Clinical Governance, and a new quality-based General Practitioner Contract[26]. These have facilitated the development of measures of quality, such as the Quality and Outcomes Framework, which provides detailed indicators across a number of conditions[27]. However the UK is unique in this regard. Elsewhere, a bottom-up approach to quality improvement has been necessary, and evidence-based tools for measuring quality of care for complex conditions are rare. Rapid measures of quality are in even shorter supply, including in the UK. Due to the pressure of day-to-day care within general practice, it can be difficult for clinicians to systematically record the necessary data on all their patients for subsequent audit. Information such as demographic data, medical history, treatments, test results and family structure is often missing[28]. General practitioners also tend to omit recording any special aspects of care or deviations from clinical guidelines due to patients' personal circumstances or subjective experiences of their illness. Furthermore, the use of special recording forms for audit exacerbates the Hawthorne effect.

This study demonstrates that the General Practice Clinical Care Interview may provide a useful assessment of the self-reported quality of care provided by Australian general practitioners to patients with chronic and complex illnesses. Our results showed that it performs slightly better psychometrically as an overall interview rating scale, than as individual disease-specific sub-scales. As an interview rating scale of clinical care across multiple chronic conditions within a general practice, it may therefore avoid one of the major problems of disease-specific approaches to chronic disease management in general practice – the problem of comorbidity. Also, it is less resource-intensive than extracting data from medical records and avoids the problems of missing or incomplete data inherent in such data extraction. Lastly, it recognises the deviations from guideline-based care based on a general practitioner's personal knowledge of the patient.

The subscales may themselves also be useful in assessing provider-reported quality of care for the three specific diseases. However, the internal consistency of the asthma and heart disease subscales was substantially lower than that of diabetes and the scale overall. This may reflect the larger proportion of very specific clinical assessment items in the diabetes sub-scale in comparison to elements for ongoing care and practice organisation in comparison with the other two sub-scales. It may also reflect the fact that greater effort has been made to codify optimal care of diabetes in general practice in the published clinical guidelines in comparison with the other two diseases.

Other studies have evaluated the utility of questionnaires to measure attitudes and activities related to preventive and clinical care [29]. To our knowledge, the GPCCI is one of the first validated rating scales of clinical care in general practice by interview. In addition, many existing measures of patient-centred care are patient-assessed [30]; whereas our scale acknowledges the importance of considering this aspect of care from a general practitioner perspective. It would be interesting to explore in more detail cases in which general practitioners did not follow a guideline and their justification for it: this is an area for further development and future research.

A number of methodological limitations of the study must be considered. In addition to the inherent problems with audits of medical records, the use of multiple raters of the patients' medical notes had the potential to increase variability. We were able to offset this limitation to some degree by demonstrating good inter-rater reliabilities, but the study would have been stronger had it been feasible for one rater to perform all the data extraction across Australia. We also plan to examine the correlation between the GPCCI and other measures of quality care especially patient-reported measures. We were also unable to quantify possible sources of error in the audit – particularly the contribution of coding errors by assessors and errors in the charts where aspects of care were not recorded.

This is only the first study of the validity of the GPCCI and the findings should be considered preliminary. Further information on psychometric properties of the GPCCI is also desirable with a larger sample of general practitioners. This will determine the underlying factor structure of the instrument, which may differ from the three disease or four processes of care components. There is also a need to further test the external validity against other measures of quality and its utility in studies of quality of care for patients with chronic disease in Australian general practice.

## Conclusion

The GPCCI may be a useful tool in assessing the quality of care of chronic disease by interview in Australian general practice. It is relatively easy to administer. This study suggests that it has good internal consistency when used as an overall scale, it has concurrent validity with patients' medical records in Australian general practice and that further evaluation of its properties, validity and utility should be carried out.

## Competing interests

The author(s) declare they have no competing interests.

## Authors' contributions

JP, FI, CA, JG, CH, TB, GPD, JB and MH made substantial contributions to conception and design of the study. JP, FI, CA, CH, TB were involved in the data collection. JP and UJ undertook the data analyses and all authors contributed to the interpretation. All authors were involved in drafting the manuscript or revising it critically for important intellectual content. All authors have given final approval of the version to be published.

## Pre-publication history

The pre-publication history for this paper can be accessed here:



## Supplementary Material

Additional File 1 Appendix I: Comparison of Guideline Recommendations and items in the GPCCI. The table provides a comparison of clinical guidelines for Asthma, Type 2 Diabetes and Ischaemic Heart Disease/Hypertension with items in the GPCCI. Click here for file

Additional File 2 Appendix II: Record Audit: Distribution of cases by practice. This table shows a breakdown of cases by practice for the record audit Click here for file

Additional File 3 Appendix III: Comparison of items and scoring for components of the GPCCI and Record Audit. The table provides a comparison of the items in the GPCCI with the items in the Medical Record Audit Click here for file
