# Supplementary material for: Quality of chronic disease care in general practice: the development and validation of a provider interview tool
Source: BMC Fam Pract. 2007 Apr 19;8:21. doi: 10.1186/1471-2296-8-21 (PMC1865546; doi:10.1186/1471-2296-8-21)
Supplement: Additional File 1 — Appendix I: Comparison of Guideline Recommendations and items in the GPCCI. The table provides a comparison of clinical guidelines for Asthma, Type 2 Diabetes and Ischaemic Heart Disease/Hypertension with items in the GPCCI. [file 1471-2296-8-21-S1.doc]

**Additional file 1**

**Appendix I: Comparison of Guideline Recommendations and items in the GPCCI.**

| **ASTHMA** | **NAC Asthma Management Handbook and 3+ Visit plan** | **GPCCI items** |
| --- | --- | --- |
| Assessment | Page 14: Assess asthma overall disease severity when patient is stable | Severity assessed |
|  | Pages 4 and 14: Spirometry should be used for diagnosis and assessment of progress | Spirometry in past 12 months |
|  | Page 20: Ensure proper use of the medication delivery device. | Review inhaler use |
|  | Pages 18-19: Assess food intolerance, Assess exercise triggers but promote full participation in exercise, Avoid smoking and passive smoking. | SNAP risk factors assessed |
| Patient education | Page 26: Education to help patients gain skills and motivation to control their asthma should be a component of all consultations. | Education for self management |
|  | 3+Plan: GP is responsible for ensuring that self management education takes place | Who provides self management education |
|  | Page 26: Education should include education on trigger factors | Education on trigger factors |
|  | 3+Plan: Involve other members of health care team such as nurses, asthma educators and pharmacists | Education referral |
| Ongoing management and practice organisation | Page14-17: Assess and achieve best lung function including assessment of high patient, use of preventive therapy, referral where appropriate. | Poor control resulting in further assessment, treatment or referral |
|  | Page 14-26 The 6 step asthma management plan  3+Plan (3 planned visits funded under the Medicare Benefits Schedule for planned care) | Planned management vs. symptom control |
|  | Page 83: Patients with moderate to severe asthma requiring care from GP and 2 other providers (MBS item) | Care plan |
|  | Page 15: Frequent visits to the ED or hospital admission in previous 12 months indicated risk of life-threatening asthma | Admitted to hospital |
|  | Page 23: Develop a written action plan for patients with asthma | Written action plan |
|  | Page 13: Level of evidence for 6 step Asthma Management Plan | Use of evidence based written guidelines |
|  | Not specifically recommended but considered useful for home monitoring and self management | Patient held record and used |
|  | Page 26: Review regularly.  3+ plan: 3 Planned visits over 12 months | Arrange follow up and attended |
|  | Not specifically recommended but considered useful for recall and monitoring. | Patient register and type |
| **DIABETES** | **RACGP Diabetes Management in General Practice 2003** | **GPCCI items** |
| Case finding | RACGP Guide based on NHMRC guidelines for early detection of diabetes 2003. | Guidelines for case finding |
|  | Page 5: Systematic and opportunistic: People at high risk for type 2 diabetes: IGT, IFG; Aboriginal and Torres Strait Islanders aged 35+; Pacific islanders, people from Indian subcontinent, Chinese aged 35+; People over 45 years with Obesity (BMI 30+)or Hypertension; Previous CVD; women with PCO who are obese. | Method of patient identification for screening |
|  | Page 5: Diagnosis based on laboratory plasma glucose measurement and confirmed on a separate day if asymptomatic. Preferred Fasting plasma glucose with OGT if between 5.6-6.9 mmol/L. | Screening and screening method |
| Assessment | Page 24: Annual Cycle of Care (also Medicare Schedule):  HbA1c once per year  BMI every 6 months  BP every 6 months  Fasting lipid once per year  Microalbumen once per year  Feet exam every 6 months  Eye exam at least every 2 years  Review smoking, healthy eating, physical activity every year | HbA1c in last 12 months |
|  |  | BMI calculated in last 6 months |
|  |  | BP in last 6 months |
|  |  | Fasting lipids in last 12 months |
|  |  | Microalbumen in last 12 months |
|  |  | Feet assessed in last 6 months |
|  |  | Eyes examined for retinopathy in 24m |
|  |  | SNAP risk factors assessed |
| Patient education | Page 24: Self care education at least yearly. | Self management education |
|  | Page 13-15: Consider referral to diabetes educator or dietician to consolidate education. | Referred for education |
| Ongoing management and practice organisation | Page 16: Targets for glycaemic control < or = 7%  Page 29: Medications for type 2 diabetes  Page 36-7: Poor control should result in reassessment, changes to treatment or referral | High HbA1c results in change in assessment, treatment, referral |
|  | Page 3: Care plans for patients requiring multidisciplinary care (also MBS) | Care planning |
|  | Page 40-54: Poor control and complications are likely to result in hospitalization | Admitted to hospital |
|  | Page 68: Based on evidence based guidelines from NHMRC | Written evidence based guidelines |
|  | 80-82: May be used as a patient held record. | Patient held record and used |
|  | Page 23: Regular follow up every 3-4 months | Follow up and attended |
|  | Page 22: A systematic approach is facilitated by a disease register | Register and type |
|  | Page 3: MBS (SIP) incentives for establishment and use of register to support annual cycle of care | Use of register for monitoring cycle of care and frequency |
| **IHD & HYPERTENSION** | **National Heart Foundation of Australia:**  **Hypertension Management Guide for Doctors 2004 (MHGD)**  **Reducing Risk in Heart Disease 2004 (RRHD)** | **GPCCI items** |
| Case finding | MHGD: Page 4-5: Important in all patients with hypertension, past history of CVD, dyslipidaemia and diabetes. | Identify patients for CV risk assessment |
|  | MHGD: Page 4-6: Assessment of CV risk be based on absolute risk of CV event using an appropriate calculator (eg NZ CR calculator) | Method of assessment |
|  | MHGD: Page 4: Factors influencing absolute CV risk are common (age, male, family history, smoking, dislipidaemia, diabetes, certain population groups) | Proportion of patients at risk identified |
| Assessment | MHGD: Monitor BP every 3-6 months depending on risk | BP in last 6 months |
|  | MHGD: Page 12: Assess fasting TC, LDL, HDL and TG yearly | Fasting lipids in last 12 months |
|  | MHGD: Page 11: Assess modifiable risk factors including smoking, diet, physical activity and alcohol  RRHD: Page 2: Assess SNAP risk factors | SNAP risk factors assessed |
| Patient education | RRHD: Page 5: All patients with CVD should be referred for secondary prevention education or cardiac rehabilitation | Self management education and who provided |
| Ongoing management & practice organization | MHGD: Page 21: For poor control: reassess for risk factors, secondary hypertension, review medications, review patient compliance, consider home monitoring, consider referral. | High BP results in change in assessment, treatment, referral |
|  | MHGD: A therapeutic plan should be implemented on all patients with hypertension | Care planning |
|  | Not specifically addressed in guidelines. However complications of hypertension (eg renal, cardiac, stroke) or secondary events are likely to result in hospitalisation | Admitted to hospital |
|  | MHGD: Page 9: In all patients consideration should be given to obtaining BP measurements outside the clinical setting either by self measurement at home or ambulatory monitoring. | Self monitoring |
|  | MHGD: Page 23: Guidelines based on systematic review and other evidence based guidelines. Distributed to all GPs in Australia  RRHD: Page 6: Source evidence based guidelines. Distributed to all GPs in Australia | Written evidence based guidelines |
|  | Not specifically mentioned in guidelines but considered important in long term follow up. | Patient held record and used |
|  | MHGD: Page 22: Long term follow up 3-6 monthly with recall if necessary | Follow up and attended |
|  | MHGD: Page 21: Recommends setting a register and recall system for long term follow up especially for non attenders. | Register, type |
