# Supplementary material for: Quality of chronic disease care in general practice: the development and validation of a provider interview tool
Source: BMC Fam Pract. 2007 Apr 19;8:21. doi: 10.1186/1471-2296-8-21 (PMC1865546; doi:10.1186/1471-2296-8-21)
Supplement: Additional File 2 — Appendix II: Record Audit: Distribution of cases by practice. This table shows a breakdown of cases by practice for the record audit [file 1471-2296-8-21-S2.doc]

**Additional File 2**

**Appendix II: Record Audit: Distribution of cases by practice**

| Practice | Asthma | | Diabetes | | IHD/Hypertension | | Total | |
| --- | --- | --- | --- | --- | --- | --- | --- | --- |
|  | N | % | N | % | N | % | N | % |
| 101 | 19 | 31 | 21 | 34 | 21 | 34 | 61 | 100 |
| 102 | 12 | 21 | 18 | 31 | 28 | 48 | 58 | 100 |
| 103 | 5 | 16 | 6 | 19 | 20 | 65 | 31 | 100 |
| 104 | 5 | 16 | 11 | 36 | 15 | 48 | 31 | 100 |
| 201 | 13 | 27 | 16 | 33 | 20 | 41 | 49 | 100 |
| 202 | 7 | 20 | 13 | 37 | 15 | 43 | 35 | 100 |
| 301 | 17 | 28 | 21 | 35 | 22 | 37 | 60 | 100 |
| 302 | 14 | 23 | 25 | 42 | 21 | 35 | 60 | 100 |
| 401 | 5 | 19 | 8 | 30 | 14 | 52 | 27 | 100 |
| 402 | 13 | 24 | 24 | 44 | 17 | 32 | 54 | 100 |

**Proportion of Asthma Cases (%) by practice with 95% confidence intervals**

**Proportion of Diabetes Cases (%) by practice with 95% confidence intervals**

**Proportion of IHD/Hypertension Cases (%) by practice with 95% confidence intervals**
