# Supplementary material for: Quality of chronic disease care in general practice: the development and validation of a provider interview tool
Source: BMC Fam Pract. 2007 Apr 19;8:21. doi: 10.1186/1471-2296-8-21 (PMC1865546; doi:10.1186/1471-2296-8-21)
Supplement: Additional File 3 — Appendix III: Comparison of items and scoring for components of the GPCCI and Record Audit. The table provides a comparison of the items in the GPCCI with the items in the Medical Record Audit [file 1471-2296-8-21-S3.doc]

**Additional File 3**

**Appendix III: Comparison of items and scoring for components of the GPCCI and Record Audit.**

| **ASTHMA** | **Record Review** | | **GPCCI** | |
| --- | --- | --- | --- | --- |
|  | **Items** | **Score** | **Items** | **Score** |
| Assessment | Severity | 2 | Severity | 1 |
|  | Spirometry/Peak flow test | 1 | Spirometry | 1 |
|  | SNAP risk factors Assessed | 1 | Review inhaler use | 1 |
|  |  |  | SNAP risk factors assessed | 1 |
| Patient education | Education for self management | 1 | Education for self management | 1 |
|  | Education on inhaler use | 1 | Who provides self management education | 1 |
|  | Education on trigger factors | 1 | Education on trigger factors | 1 |
|  |  |  | Education referral | 1 |
| Ongoing management and practice organisation | Night cough or wheeze resulting in medication change | 1 | Poor control resulting in further assessment, treatment or referral | 2 |
|  | Written action plan | 1 | Planned management vs. symptom control | 2 |
|  | Admitted to hospital in past 12 m for asthma | 1 | Care plan | 3 |
|  | Recall or review date in patient record | 1 | Admitted to hospital in past 12 months | 1 |
|  |  |  | Written action plan | 1 |
|  |  |  | Use of written guidelines | 1 |
|  |  |  | Patient held record and used | 2 |
|  |  |  | Arranged follow up and patient attended | 2 |
|  |  |  | Patient register and type | 2 |
| Total |  | 11 |  | 24 |

| **DIABETES** | **Record Review** | | **GPCCI** | |
| --- | --- | --- | --- | --- |
|  | **Items** | **Score** | **Items** | **Score** |
| Case finding | Diagnosed on the result of screening | 1 | Guidelines for case finding | 1 |
|  |  |  | Method of patient identification for screening | 2 |
|  |  |  | Screening and screening method | 3 |
| Assessment | HbA1c in last 6 months | 1 | HbA1c in last 12 months | 1 |
|  | BMI calculated in last 6 months | 1 | BMI calculated in last 6 months | 1 |
|  | BP in last 6 months | 1 | BP in last 6 months | 1 |
|  | Fasting lipids in last 12 months | 1 | Fasting lipids in last 12 months | 1 |
|  | Microalbumen in last 12 months | 1 | Microalbumen in last 12 months | 1 |
|  | Feet assessed in last 6 months | 1 | Feet assessed in last 6 months | 1 |
|  | Eyes examined for retinopathy in 24m | 1 | Eyes examined for retinopathy in 24m | 1 |
|  | SNAP risk factors Assessed | 1 | SNAP risk factors assessed | 1 |
| Patient education | Self management education in past 12m | 1 | Self management education | 1 |
|  |  |  | Referred for education | 1 |
| Ongoing management and practice organisation | If high HbA1c change in assessment, treatment or referral | 1 | High HbA1c results in change in assessment, treatment, referral | 2 |
|  | If high BP change in assessment, treatment or referral | 1 | Care planning | 2 |
|  | Recall or review | 1 | Admitted to hospital in past 12 m | 1 |
|  | Admitted to hospital in past 12 m for diabetes | 1 | Written evidence based guidelines | 1 |
|  |  |  | Patient held record and used | 2 |
|  |  |  | Arranged follow up and patient attended | 2 |
|  |  |  | Register and type | 2 |
|  |  |  | Use of register for monitoring cycle of care and frequency | 2 |
| Total |  | 14 |  | 30 |

| **IHD & Hypertension** | **Record Review** | | **GPCCI** | |
| --- | --- | --- | --- | --- |
|  | **Items** | **Score** | **Items** | **Score** |
| Case finding | Diagnosed on the result of screening | 1 | Identify patients for CV risk assessment | 1 |
|  |  |  | Method of identification and assessment | 2 |
|  |  |  | Proportion of patients at risk identified | 1 |
| Assessment | BP in last 6 months | 1 | BP in last 6 months | 1 |
|  | Fasting lipids in last 12 months | 1 | Fasting lipids in last 12 months | 1 |
|  | SNAP risk factors assessed | 1 | SNAP risk factors assessed | 2 |
| Patient education | Provided or referred for self management education in past 12m | 1 | Self management education and who provided | 2 |
| Ongoing management & practice organization | If high BP change in assessment, treatment or referral | 1 | High BP results in change in assessment, treatment, referral | 2 |
|  | If high Lipids change in assessment, treatment or referral | 1 | Care planning | 3 |
|  | Recall or review | 1 | Admitted to hospital in past 12 m | 1 |
|  | Admission in past 12 m for IHD/Hypert | 1 | Self monitoring | 1 |
|  |  |  | Written evidence based guidelines | 1 |
|  |  |  | Patient held record and used | 2 |
|  |  |  | Arranged follow up and patient attended | 2 |
|  |  |  | Register, type | 2 |
| Total |  | 9 |  | 24 |

**Summary of scoring for Record Audit and GPCCI**

| Components | Asthma | | Diabetes | | IHD/Hypertension | |
| --- | --- | --- | --- | --- | --- | --- |
|  | Audit | GPCCI | Audit | GPCCI | Audit | GPCCI |
| Case finding | 0 | 0 | 1 | 6 | 1 | 4 |
| Assessment | 4 | 4 | 8 | 9 | 3 | 4 |
| Patient education | 3 | 4 | 1 | 2 | 1 | 2 |
| Ongoing management and practice organisation | 4 | 16 | 4 | 13 | 4 | 14 |
| Total | 11 | 24 | 14 | 30 | 9 | 24 |

**Descriptive statistics for Record Audit and GPCCI sub-scores**

|  | Record Audit | | | GPCCI | | |
| --- | --- | --- | --- | --- | --- | --- |
|  | Mean | St Dev | Skewness | Mean | St Dev | Skewness |
| Asthma | 4.7 | 1.72 | 0.83 | 10.5 | 4.1 | -0.55 |
| Diabetes | 9.3 | 2.71 | -0.71 | 18.0 | 4.5 | -1.19 |
| IHD/ Hypertension | 5.4 | 1.35 | -0.087 | 12.0 | 3.3 | -0.39 |
